# Negative causal exploration of systemic sclerosis: a Mendelian randomization analysis

**DOI:** 10.1038/s41598-024-55808-w

**Published:** 2024-03-03

**Authors:** Zesen Han, Peisen Han, Fang Wang, Huayu Zheng, Xiujian Chen, Hongyu Meng, Fenglei Li

**Affiliations:** 1 Hua Country People’s Hospital, Anyang, 456400 Henan Province China; 2 The Department of Computer and Information Engineering, Henan University, Kaifeng, 475001 China

**Keywords:** Systemic sclerosis, Thyroid disease, Appendicitis, Mendelian randomization, FinnGen, Computational biology and bioinformatics, Immunology, Medical research, Rheumatology

## Abstract

Systemic sclerosis (SSc), also known as scleroderma, is an autoimmune-related connective tissue disease with a complex and unknown pathophysiological mechanism with genes association. Several articles have reported a high prevalence of thyroid disease in SSc patients, while one study suggested a potential contribution of appendicitis to the development of SSc. To investigate this causal association, we conducted Mendelian randomization (MR) analysis using instrumental variables (IVs) to assess exposure and outcome. In the MR study involving two cohorts, all analyses were conducted using the TwoSampleMR package in R (version 4.3.0). Single nucleotide polymorphisms (SNPs) meeting a statistically significant threshold of 5E−08 were included in the analysis. Multiple complementary approaches including MR-IVW, MR-Egger, weighted median, simple mode, and weighted mode were employed to estimated the relationship between the exposure and outcome. Leave-one-out analysis and scatter plots were utilized for further investigation. Based on the locus-wide significance level, all of the MR analysis consequences manifested no causal association between the risk of appendicitis with SSc (IVW OR 0.319, 95% CI 0.063–14.055, *P* = 0.966). Negative causal effects of autoimmune thyroiditis (AT) on SSc (IVW OR 0.131, 95% CI 0.816–1.362, *P* = 0.686), Graves’ disease (GD) on SSc (IVW OR 0.097, 95% CI 0.837–1.222, *P* = 0.908), and hypothyroidism on SSc (IVW OR 1.136, 95% CI 0.977–1.321, *P* = 0.096) were derived. The reverse MR revealed no significant causal effect of SSc on thyroid disease. According to the sensitivity analysis, horizontal pleiotropy was unlikely to distort the causal estimates. The consequences indicated no significant association between AT, GD, and hypothyroidism with SSc. Similarly, there was no observed relationship with appendicitis.

## Introduction

Systemic sclerosis (SSc) is an autoimmune-related connective tissue disease, also known as scleroderma, which affect the skin and internal organs. The intricate pathophysiological mechanisms and the early endothelial damagecontribute to a higher risk of morbidity and mortality in patients with SSc, desipe their outward appearance resembling that of a “Panda”^1^, particularly among children and adolescents^2^. The resulting fibrotic reaction, driven by inflammatory infiltrates, had led to therapeutic concepts being developed based on advancements in genetic technology^3^.

Concentrating on the pathogenetic pathways, vasculopathy may play a pivotal role throughout the entire spectrum of clinical manifestations^4^. Early organ damage and lack of disease-modifiying therapies contributes to increased morbidity and mortality, and diminished quality of life.^5^ Despite its low prevalence, SSc imposes a substantial burden on healthcare costs, unemployment rates, and productivity losses.^6^ Over the past decade, there has been growing interest in exploring the interplay between pathology and disease development, encompassing factors such as environmental influences and genetic predisposition. Moreover, various disease have been implicated as potential triggers for SSc, for instance, appendicitis has been associated with an increased risk of occurrence and progression^7^. Additionally, investigators have reported a causal relationship between SSc and thyroid disease including autoimmune thyroiditis (AT), Graves’ disease (GD), and hypothyroidism^8^. Are these truly causative factors? And does thyroid dysfunction indeed play a role in SSc or vice versa?

Up to date, the etiology of SSc remains unclear, however, evidence suggests that genetic factors may play a significant role in triggering the disease^9^. In 2017, Chairta et al. conducted a Meta-analysis which revealed frequent associations between specific alleles in the HLA-DRB1, HLA-DQB1, HLA-DQA1, and HLA-DPB1 genes as well as variants in STAT4, IRF5 and CD247 with SSc. NFKB1, CSF3R, STAT4, IFNG, PRL and ILs are key components of the interaction network involving non-HLA genes associated with SSc^10^. The candidate gene approach (CGA) and genome-wide association study (GWAS) are fundamental methods employed for genetic association studies. These studies have successfully identified associations between genetic polymorphisms and SSc patients^11^, potentially targeting candidate genes by approved medications for immune-mediated diseases^12^.

The Mendelian randomization (MR) approach has proven to be a powerful tool in establishing causal associations between exposures and outcomes in various fields of research. By leveraging the natural randomization provided by genetic variation, MR studies have the ability to reduce the impact of confounding biases that often plague observational studies. One of the key aspects of MR is its reliance on single-nucleotide polymorphisms (SNPs)—genetic variants that occur at a single position in the genome—to assess the causal relationship between an exposure and an outcome. SNPs can be thought of as the building blocks of genetic variation, and they provide a wealth of information about the role of different genes in various biological processes.

In order to conduct MR studies, researchers often follow the STROBE-MR guidelines, which provide a standardized framework for conducting MR research. These guidelines include the use of instrumental variable frameworks, 1-sample and 2-sample MR studies, among other methodologies.

Instrumental variables (IVs), as known SNPs, are particularly useful in MR studies because they allow researchers to establish a causal relationship between an exposure and an outcome by using a variable that is both predictive of the exposure and independent of any other factors that might influence the outcome. This helps to reduce the risk of confounding biases that can arise from observational studies.

1-sample and 2-sample MR studies are also crucial components of the STROBE-MR guidelines^13^. Both approaches have their own strengths and weaknesses, and the choice of which approach to use often depends on the specific research question being investigated.

The SNPs were also utilized to investigate the association between thyroid disease and other diseases. *QRFPR* rs7679475 may exert an influence on the susceptibility of AT in patients with type 1 diabetes in Chinese Han population^14^. Hypothyroidism significantly increases the incidence of non-alcoholic fatty liver disease (NAFLD), while hyperthyroidism may act as a risk factor for NAFLD through MR analysis^15^ and so forth.

The prevailing brief is that MR research has reached a state of maturity. Limited knowledge exists regarding the confident genetic links association with SSc. Additionally, clinical observations have indicated that some individuals affected by SSc have family members who experience dysfunction in their thyroid gland. The objective of the this study was to especially investigate the etiology of SSc through MR analysis.

## Methods

### Data sources

The publicly available DF9 version of the FinnGen databases (https://www.finngen.fi/), released on May 11 2023, encompasses a total of 20,175,454 variants and includes data from 210,870 females and 166,407 males across 2,272 endpoints^16^.

The phenocode of SSc in FinnGen was “M13_SYSTSLCE”, which included 619 cases compared to 365,533 samples in the control group. The phenocode for appendicitis was “APPENDACUT_COMPLIC” with 6747 cases versus346283 controls. Regarding thyroid disease, the phenocode for AT was designated as “E4_THYROIDITAUTOIM”, while GD strictly adhered to the definition of “E4_GRAVES_STRICT”, and hypothyroidism was labeled as “E4_HYTHY_AI_STRICT”. These encompassed a total of 489, 2836, and 40,926 cases respectively.

### Statistical analysis and selection of instrumental variables

The analysis was conducted using the TwoSampleMR package of R (version 4.3.0). The independent SNPs strongly associated with exposure were selected based on a *p*-value less than 5E−08. This selection was performed by applying a linkage disequilibrium (LD) threshold of R^2^ < 0.001 and a window size of 10,000 kb for clumping. The presence of statistically significant exposure SNPs (*P* < 0.05) and the absence of statistically significant outcome-related SNPs (*P* ≥ 0.05) should be ensured in order to exclude the confounders from MR studies. Additionally, the datasets of exposure and outcome were harmonized to ensure uniform effect alleles. Based on above, the most appropriate genetic instrumental variables (IVs) for initiating the MR analysis were identified.

The subsequent step involved employing multiple complementary approaches (MR-IVW, MR-Egger, weighted median, simple mode, and weighted mode) to estimate the effects of the relationship between the exposure and outcome. It is crucial to consider the heterogeneity evaluated by Cochrane Q test when *p* < 0.05 in order to utilize the IVW test effectively. Additionally, the authors conducted leave-one-out analysis to ensure reliability and consistency.

### Ethical approval

This study conformed to the principles of the Declaration of Helsinki. This is a retrospective study. All of the data came from the FinnGen datasets published online. This study has been approved by the Ethics Committee of Hua Country People’s Hospital (ID: 2023002).

## Results

### Effect of appearance on SSc

After conducting LD analysis, 10 out of 620 items with *P* values were less than 5E−08, which were considered to have a relationship among the IVs in appendicitis. Subsequently, researchers selected the SNPs that were also present in SSc and merged them together. Excluding those SNPs that showed statistical significance with the outcome variable. Finally, the authors obtained 10 SNPs for evaluation in the MR research. The resulting SNPs were rs10849448, rs149773, rs200540616, rs201768, rs2348650, rs3738182, rs707958, rs7697491, rs78817037, and rs976568 (Supplemental Table 1).

The procedure excluded the SNPs of rs2348650, rs7697491 due to their palindromic nature and intermediate allele frequencies. The remaining SNPs were involved in the computation.

**Figure 1:**
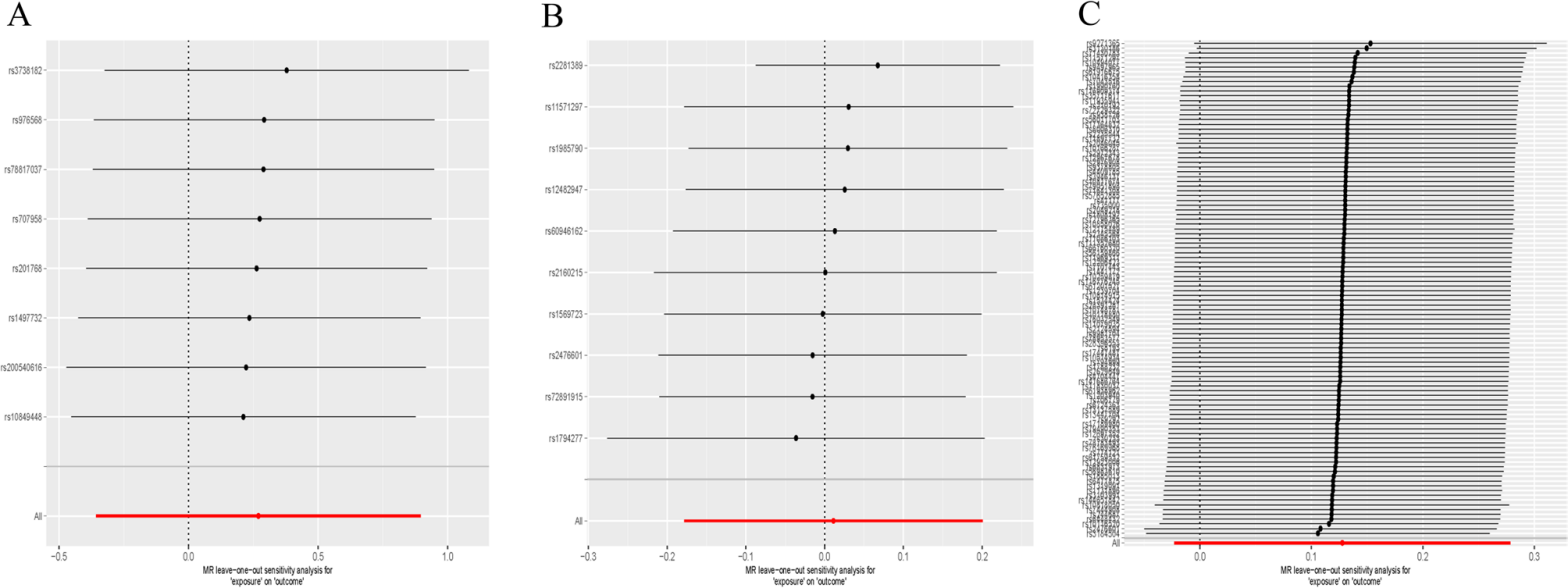
Leave-one-out plots of mendelian randomization analysis. (**A**) Causal estimates for appendicitis on SSc. (**B**) Causal estimates for GD on SSc. (**C**) Causal estimates for hypothyroidism on SSc.

**Figure 2:**
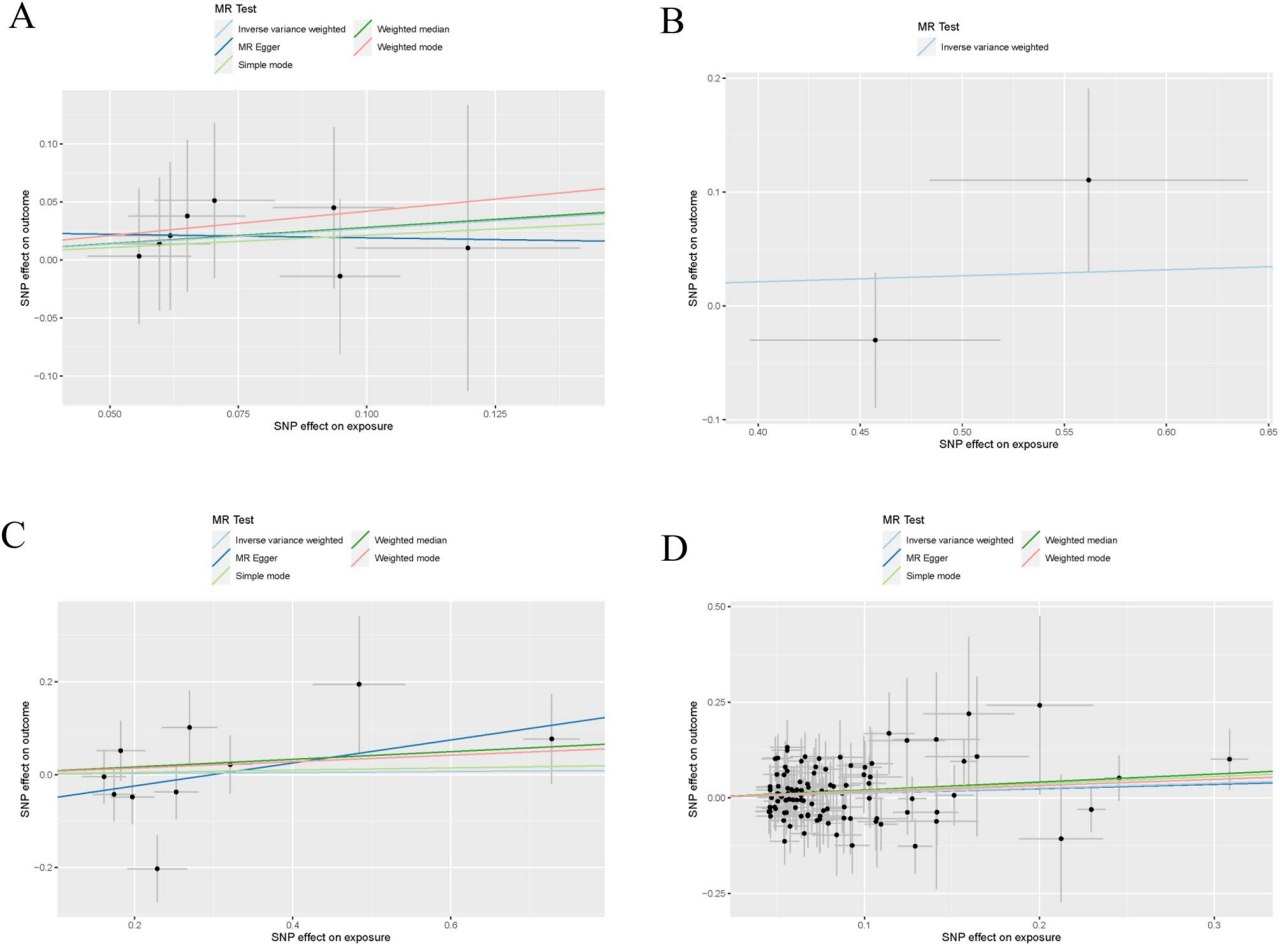
Scatter plots of the causal relationships with different MR methods. (**A**) Causal relationship of appendicitis on SSc. (**B**) Causal relationship of AT on SSc. (**C**) Causal relationship of GD on SSc. (**D**) Causal relationship of hypothyroidism on SSc.

The observed consequences did not demonstrate any causal association between the occurrent risk of appendicitis and SSc (IVW OR 0.319, 95% CI 0.063–14.055, *P* = 0.966) (Table 1), which deviated from the reported online finding. Meanwhile, the MR-Egger regression analysis revealed no evidence of horizontal pleiotropy. And there was no significant heterogeneity (*P* > 0.05) (Table 2). The leave-one-out analysis demonstrated stability even after excluding one SNP at a time (Fig. 1A), while the scatter plots presented in Fig. 2A.

**Table 1:** The association consequences of the MR analysis.

Exposure	Outcome	Method	Nsnp	OR	95% CI	*P* values
Appearance	SSc	MR Egger	8	0.940	(0.063, 14.055)	0.966
		Weighted median	8	1.322	(0.630, 2.783)	0.462
		Inverse variance weighted	8	1.310	(0.700, 2.448)	0.398
		Simple mode	8	1.236	(0.423, 3.616)	0.710
		Weighted mode	8	1.520	(0.519, 4.451)	0.470
AT	SSc	Inverse variance weighted	2	1.054	(0.816, 1.362)	0.686
GD	SSc	MR Egger	10	1.281	(0.853, 1.923)	0.267
		Weighted median	10	1.086	(0.891, 1.323)	0.414
		Inverse variance weighted	10	1.011	(0.837, 1.222)	0.908
		Simple mode	10	1.024	(0.740, 1.418)	0.888
		Weighted mode	10	1.072	(0.844, 1.361)	0.582
Hypothyroidism	SSc	MR Egger	104	1.122	(0.817, 1.539)	0.479
		Weighted median	104	1.232	(0.964, 1.574)	0.095
		Inverse variance weighted	104	1.136	(0.977, 1.321)	0.096
		Simple mode	104	1.206	(0.699, 2.080)	0.503
		Weighted mode	104	1.177	(0.883, 1.567)	0.269
SSc	AT	Inverse variance weighted	2	0.975	(0.430, 2.211)	0.952
SSc	GD	Inverse variance weighted	2	1.387	(0.953, 2.019)	0.087
SSc	Hypothyroidism	Inverse variance weighted	2	1.028	(0.801, 1.320)	0.828

SSc: systemic sclerosis; AT: autoimmune thyroiditis; GD: graves’ disease.

**Table 2:** All of the heterogeneity and horizontal pleiotropy test of the MR analysis.

Exposure	Outcome	Methods	Heterogeneity		Horizontal pleiotropy	
			Q	*P* values	MR-Egger intercept	*P* values
Appearance	SSc	MR Egger	0.780	0.993	0.102	0.813
		Inverse variance weighted	0.841	0.997		
AT	SSc	MR Egger	NA	NA	NA	NA
		Inverse variance weighted	1.854	0.173		
GD	SSc	MR Egger	11.854	0.158	-0.074	0.237
		Inverse variance weighted	14.271	0.113		
Hypothyroidism	SSc	MR Egger	74.652	0.981	0.001	0.927
		Inverse variance weighted	74.661	0.984		
SSc	AT	MR Egger	NA	NA	NA	NA
		Inverse variance weighted	13.011	0.0003		
SSc	GD	MR Egger	NA	NA	NA	NA
		Inverse variance weighted	16.506	4.85e−05		
SSc	Hypothyroidism	MR Egger	NA	NA	NA	NA
		Inverse variance weighted	83.690	5.79e−20		

SSc: systemic sclerosis; AT: autoimmune thyroiditis; GD: graves’ disease.

### Influence of thyroid disease with SSc

In the MR analysis, three types of thyroid disease were included, namely AT, GD, and hypothyroidism. No significant genetic SNP was identified, therefore the researchers took into account the potential implication of choric thyroiditis. We are inclined to attribute the causality to SSc individually for each type of thyroid disease. Initially, our focus was on AT where two independent IVs with R^2^ values less than 0.001and a total of 319 significant SNPs (*P* < 5E−08) contributed to the outcome. That the inclusion of rs6679677 and rs9271365 (Supplemental Table 2) as IVs led us to employ only the IVW method on the MR analysis, (OR 0.131, 95% CI 0.816–1.362, *P* = 0.686) (Table 1). Heterogeneity was not observed as indicated bya *P* value of 0.173 without heterogeneity test results available at hand (Table 2). Unfortunately, we were unable to obtain analytical results for pleiotropy testing or leave-one-out plot generation. Instead, scatter plots were examined in Fig. 2B.

Second, we conducted an investigation into the association between GD and SSc, resulting in the selection of 13,119 meaningful SNPs after comparison to a significance threshold of 5E-08. Considering clump analysis, fifteen SNPs were chosen as IVs. The SNP rs61734579 was excluded from the analysis due to its lack of statistical significance (*P* < 0.05) (Supplemental Table 3). Additionally, four SNPs (rs11038350, rs12199670, rs145008938, and rs56738967) were removed from the MR analysis to avoid repetition based on allele frequencies. The MR analysis revealed no causal relationship between GD and SSc (IVW OR 0.097, 95% CI 0.837–1.222, *P* = 0.908) (Table 1), with no significant evidence of heterogeneity or pleiotropy observed (*P* > 0.05 for both) (Table 2). The leave-one-out plot is presented in Fig. 1B and scatter plots are displayed in Fig. 2C.

The previous study focused on the association between hypothyroidism and SSc. The *P* values of 25,854 clauses were inferior to 5E-08. After applying LD analysis, only 131 SNPs remained among these clauses. Further filtration was performed by excluding significant items from the IVs, resulting in a total of 118 SNPs for subsequent analysis. (Supplemental Table 4) Ultimately, we observed a lack of negative influence as indicated by the *P* value of IVW of 0.096 which is greater than the threshold of 0.05 consistent with other methods’ conclusions (Table 1). Additionally, no heterogeneity or pleiotropy was detected (Table 2). The leave-one-out plot is exhibited in Fig. 1C and scatter plots are held up in Fig. 2D.

The interaction design for the MR analysis of thyroid disease with SSc aimed to modify the status of whether the SSc is a cause or a result. We positioned SSc as the exposure variable and probed it with the IVs. Out of 661 SNPs identified before LD, only two (rs732163 and rs1794269) (Supplementary Table 1) were found to be suitable as IVs. It was deemed necessary to perform separate analysis for different subtypes of thyroid disease. The MR report illustrated that there was no discernible contribution from SSc to AT, GD, and hypothyroidism. All P values obtained using the IVW method were superior to 0.05. as detailed in Table 1. Due to limited SNP availability, other methods could not be employed in the MR calculation. Additionally, attempting heterogeneity assessment based on IVW yielded inconclusive results (Table 2). Unfortunately, we were unable to conduct pleiotropy tests (Table 2), which led us to abandon generating leave-one-out plots and scatter plots due to poor performance.

## Discussion

After conducing the MR analysis, we found no causal association between thyroid disease and SSc indicting that SSc is unlikely to be inherited due to the investigation extending to ancestors who were never attacked by the same disease except for thyroid.

In a study conducted at a single Israeli center, no new cases of AT were discovered amony fifty patients with SSc, despite ten patients having concurrent thyroid disease^17^. This finding supports the hypothesis that SSc does not contribute to the development of AT. A systematic review and meta-analysis alos revealed no association between GD or hyperthyroidism and SSc. But, it did identify a significantly higher prevalence of AT in individuals with SSc^18^.

Whereas, numerous articles have been conducted on the exhibition of various types of thyroid disease and SSc. In the early 2007, three cases of GD were observed in female patients with SSc compared to none in the control group, demonstrating statistical significance (*P* = 0.0140). The authors recommended including thyroid function testing as part of clinical laboratory work for patients with SSc^19^. In the same year, Professor Marasini B focused on whether SSc increased the incidence of thyroid dysfunction but concluded that while it may not exacerbate such conditions, antithyroid peroxidase antibodies could play a role^20^. Over time, research on this topic has continued to advance. Japanese researchers identified a higher prevalence of hypothyroidism among individuals with autoimmune thyroid disorders within a total sample size of 210 patients with both conditions^21^. Another study found a greater occurrence of mild hypothyroidism among female patients with SSc^22^.

A nationwide population-based cohort study was conducted to address concerns regarding the worse prognosis observed among individuals diagnosed with both diseases. It is believed that dysfunction may contribute to decrease survival rates in patients with SSc^23^. The concept of AT in SSc has been extensively investigated, yielding similar findings^24^.

Recently, αvβ3 integrin has been identified as a crucial link between fibrosis development and thyroid hormones (THs) in SSc. The finding suggest that the integrin exerts its function by deregulating THs binding site regulation^25^. Previous studies have demonstrated elevated levels of the Th1 chemokine CXCL10 in serum or tissue, which may play a key factor in systemic rheumatologic diseases^26^. In 2022, IL-10 was discovered to be increased in patients with Hashimoto's thyroiditis and SSc, indicating its significance as an indicator of Breg cells’ functionality^27^.

As for the pathogenesis of appendicitis on SSc, only one study has been conducted to date^8^, and conflicting evidence from MR analysis exists. Further trials are required to confirm this association.

Thus far, no definitive causal factors linked to SSc have been identified except for having at least one first-degree relative diagnosed with an autoimmune disease being considered a potential risk factor for its development^28^. However, we have excluded thyroid disease including AT, GD, and hypothyroidism as primary causes. Additionally, appendicitis has been ruled out as a contributing factor. The exploration into the etiology of SSc will continue through various avenues such as exploring environmental factors.

The Mendelian randomization analysis borrowed economic technical statistics to address the challenges in epidemiology and human biology^29^ effectively bridging the gap between genetic association and observational summary data, such as GWAS^30^. This approach successfully mitigated biases arising from confounding or reverse causality^31^. Furthermore, it is important for investigators to consider not only positive influences but also negative consequences when formulating guidelines. For example, although systemic inflammatory regulators were not identified as a causal risk factor for Alzheimer's disease^32^, increased coffee consumption in women was not found to be association with breast cancer risk^33^, and no evidence supported an association between the inflammatory bowel disease with the atrial fibrillation^34^. While this article may not directly establish causal relationships for certain diseases, it highlights the importance of excluding interfering factors when exploring underlying causes in order to achieve accurate conclusions. The analytical implications of Mendelian randomization did not consistently align with those of other types of trials. A meta-analysis was conducted on 12,218 samples from 10 population cohorts, revealing an increased risk of lung cancer in patients with SSc. However, the findings from the MR study did not support the association specifically with lung adenocarcinoma, squamous cell lung carcinoma, or small-cell lung cancer^35^. Similarly, a meta-analysis involving 1448 SSc patients demonstrated a lower left ventricular ejection fraction (LVEF) compared to controls. The observation suggested a causal correlation between SSc and LVEF (OR = 0.9966, 95% CI 0.9935–0.998, *P* = 0.0398). However, no confirmed genetic link between SSc and left ventricular end-diastolic volume or left ventricular mass was found due to the lack of significant statistical difference^36^, which differed from previous meta-analysis. Observational epidemiological studies have proven to be unreliable indicators for causal exploration. Therefore MR analysis provides more reliable evidence regarding interventions that can produce health benefits^37^.

Causal exploration has been attempted to assess the impact of body fat distribution factors such as body mass index (BMI), waist-to-hip ratio (WHR) and WHR adjusted for BMI (WHRadjBMI) on the development of SSc. The research indicted no causative genetic correlation between any of these factors and SSc. Nevertheless, further studies are needed to rule out obesity due to limited statistical efficacy in GWAS^38^. Associations between musculoskeletal system and connective tissue disease (MSCTD) including rheumatoid arthritis (RA), Sjogren syndrome (SS), systemic lupus erythematosus (SLE), SSc, dermatomyositis (DM), polymyositis (PM), osteoarthritis (OA) of hip or knee, and ankylosing spondylitis (AS) with breast cancer (BC) have been investigated in European populations and East Asian populations based on MR analysis^39^. The analysis revealed an increased risk of rheumatoid arthritis (RA) and ankylosing spondylitis (AS) associated with birth control use in the European population, while multiple sclerosis combined with thyroid disease (MSCTD) showed an increased risk specifically with estrogen receptor-negative birth control in Europeans. Conversely, RA and systemic lupus erythematosus (SLE) demonstrated a decreased risk associated with birth control use in East Asians. Unfortunately, no relationship was reported between SSc with BC. Over the years, MR analysis has focused on elucidating the etiology of Autoimmune diseases (ADs) by exploring potential assumptions between exposure factors and disease or other outcomes, providing a novel method^40^.

In addition to other diseases, blood metabolites such as glycerol 2-phosphate for type 1 diabetes, hexadecanedioate, phenylacety-lglutamine and laurylcarnitine for RA, glycine and arachidonate for Crohns’s disease may serve as biomarkers to identify multiple ADs^41^. These findings provide further avenues of investigation into the causes of SSc by considering three types of variables: exposures, mediators, and an outcome^42^. MR mediation methods require certain assumptions that can enhance causal inference in mediation analysis^43^. In the future, it may be feasible to extend the MR approach with transcripts and metabolites^44^.

The pathogenesis exploration results of MR analysis in SSc differed from the clinical observations in thyroid diseases (AT, GD, and hyperthyroidism) and appendicitis. This phenomenon is intricate, and MR analysis can serve as an analytical approach to assess the causality of an observed association between a modifiable exposure or risk factor and a clinically relevant outcome when randomized controlled trials are not feasible for examining causality, and observational studies may yield biased associations due to confounding or reverse causality^31^. The researchers hope the further investigate the causes of SSc.

### Supplementary Information

Supplementary Tables.

## Data Availability

The datasets generated and analysed during the current study are available in the FinnGen repository, https://www.finngen.fi/ or from the corresponding author on reasonable request.
